# Nutritional, Biochemical, and Functional Properties of Pearl Millet and *Moringa oleifera* Leaf Powder Composite Meal Powders

**DOI:** 10.3390/foods13050743

**Published:** 2024-02-28

**Authors:** Faith Sibanda, Victoria A. Jideani, Anthony O. Obilana

**Affiliations:** Department of Food Science and Technology, Bellville Campus, Cape Peninsula University of Technology, Cape Town 7535, South Africa; faithsibanda73@gmail.com (F.S.); jideaniv@cput.ac.za (V.A.J.)

**Keywords:** pearl millet, *Moringa oleifera*, fermentation, malting, compositing, optimisation, mixture design

## Abstract

This study sought to improve pearl millet’s nutritional, functional, and biochemical properties through malting and fermentation. *Moringa oleifera* leaf powder (MLP) was used as a fortificant. Mixture design was used to find optimal proportions for each component that yielded a high protein and or low saturated fat content. Twelve mixtures with varying ratios of fermented and malted pearl millet flour ranging between 30–65% and MLP between 5–15% were generated through I-Optimal mixture design. The mixtures were wet-cooked, freeze-dried, and analysed for protein and fat content. The data obtained were fitted to a linear mixture model, and the search for the optimum was conducted using Numerical Optimisation for maximising protein and minimising saturated fat. The linear model was suitable for explaining total protein and saturated fat variation with r^2^ of 0.50 and 0.51, respectively. Increasing MLP increased protein content. Two final formulations, Optimisation Solution 1 (OS1) and Optimisation Solution 2 (OS2), were generated through the optimisation process. Pearl millet’s protein content increased by up to 22%, while saturated fat decreased by up to 13%; ash content increased by 75%. Polyphenol content and oxygen radical absorbance capacity increased by 80% and 25%, respectively. Final and peak viscosity were reduced by 90% and 95%, respectively.

## 1. Introduction

Pearl millet (*Pennisetum glaucum*) is widely cultivated in arid and semi-arid regions, characterised by low rainfall and infertile soils, in which other major cereals fail to yield significant harvests [1]. In South Africa, pearl millet (PM) is cultivated in the Free State, Limpopo and KwaZulu-Natal provinces. Pearl millet grains are a good source of proteins, vitamins, minerals, and energy comparable to and even superior to other major cereals’ nutrient content [2]. The protein content of pearl millet ranges between 9.4 to 11.8 g/100 g with an amino acid profile predominantly comprising the essential leucine, isoleucine, valine, and phenylalanine but lacking in lysine and methionine [3,4]. The grain also contains substantial phenolic compounds, making it a potent source of antioxidants for populations that consume the cereal in porridge or beverage formats [3,5].

Malting, fermentation, or enzymatic treatment have been suggested to improve PM’s nutritional, biochemical, and functional properties (due to protein and carbohydrate structural modifications) [6,7]. These processing techniques assist in producing lower-viscosity PM food products with reduced content of antinutritional factors and improved nutrient bioavailability. These benefits are carried over to the consumers, including weaning-stage infants, patients, or even anyone (for example, athletes) needing semi-liquid or low-viscosity meals [6,7,8,9,10].

To curb protein-energy malnutrition, PM has been combined with legumes, a concept known as compositing, which involves combining two different nutrient sources to produce a more nutrient-balanced product. However, legumes present challenges such as anti-nutritional factors, poor digestibility, toxic components, and raffinose family oligosaccharides (RFOs) that cause flatulence. Moreover, native legume proteins commonly show low stability and solubility under intensive heat treatment, which is required to achieve palatability in legume foods [11,12]. Furthermore, there is a need to address micronutrient deficiencies, which are widespread in Eastern and Southern Africa [13].

*Moringa oleifera*, cultivated in the tropics and subtropics, is a highly valued tree legume in the medical and food sectors. Moringa leaf powder (MLP) presents an excellent opportunity for improving the nutritional quality of PM-based foods as it is high in protein and the essential amino acid lysine (deficient in PM), accompanied by an exceptional overall nutrient balance [14,15]. Moreover, MLP offers substantial phenolic compounds or phytochemicals with nutraceutical functionalities such as anti-cancer, anti-tumour, anti-inflammatory, anti-hyperglycemic, and hepatoprotective in PM foods [16,17]. On the other hand, there has been extensive research on the acute and chronic toxicity of the consumption of *Moringa oleifera* leaves and leaf extracts in the nutraceutical sector. Although somewhat indicative outcomes have been acquired in mice, the safety of *Moringa oleifera* consumption in humans is inconclusive, with some studies indicating a single dose of 50 g of whole leaf powder or 320 g uniformly distributed doses over 40 days as safe [18,19,20].

Various studies have explored *Moringa oleifera*’s potential to improve the nutritional composition of pearl millet foods, mainly using *Moringa oleifera* seeds, leaves, and leaf extracts, yielding positive outcomes [21,22]. This study employed a synergistic approach to improve PM’s nutritional and functional properties through processing (malting and fermentation) and the addition of MLP. For optimum results in compositing, response surface methodology using mixture design was used to find optimal proportions for each component that yields high protein or low saturated fat content.

## 2. Materials and Methods

### 2.1. Materials

Pearl millet grains were acquired from AGT Foods, Cape Town, South Africa. Moringa leaf powder was purchased from SupaNutri Pty (Ltd.) (Graaf-Reinet, South Africa). All chemicals were purchased from Merck (Pty) Ltd. (Cape Town, South Africa).

### 2.2. Malting and Fermentation of Pearl Millet

Malting was carried out by cleaning and soaking PM grains at a 1:1.5 (*w*/*w*) ratio of grain and 0.03% caustic soda solution (to aid enzyme activity). Soaking/steeping was conducted at temperatures of 22 to 28 °C for 3 h with periodic agitation before draining the caustic solution and washing off the residue. The steeped PM grains were spread on perforated plastic trays lined with a muslin cloth, packed onto a trolley with perforated pans, and loaded into a proofing oven (Prover, Macadams, Cape Town, South Africa) at 30 °C and ~98% relative humidity. The germination occurred for 36 h before kilning (drying) in a cabinet drier (Geiger & Klotzbucher, Cape Town, South Africa) at 50 °C for 48 h. Dry germinated grains were milled with a hammer mill (TRF 400, Metalurgica Trapp, Jaraguá do Sul, SC, Brazil) and sieved through a 2 mm mesh, and the flour was packed and sealed in ziplock polypropylene bags.

Fermentation was carried out as prescribed by Osman [23] with slight modifications. Spontaneous fermentation was initiated by mixing a small batch of raw PM flour with distilled water (1:2 *w*/*v*) and incubating it at 37 °C for 24 h. A bulk fermentation slurry was then prepared using the same ratios of PM flour to water, to which a 5% portion of the fermented slurry was added as a starter culture. This process is referred to as back-slopping, and it provides active LABs to initiate bulk fermentation while shortening the time required to reach the desired pH or acidity [23,24]. The bulk fermentation was then carried out by incubating the inoculated slurry at 37 °C for 36 h before freeze-drying the paste and packaging the flour in sealed ziplock polypropylene bags.

### 2.3. Mixture Design, Modelling and Optimisation Using Numerical Optimisation

Twelve mixtures with varying ratios of fermented pearl millet flour (FPMF) and malted pearl millet flour (MPMF) within a range of 30–65%, and moringa leaf powder (MLP) ranging between 5–15% were generated through I-Optimal mixture design. FPMF and MPMF were blended, wet-cooked, and cooled to <50 °C before the addition of MLP and freeze-drying of the final pastes. The dry samples were analysed for protein and fat content.

The data from the analysis of the twelve mixtures were fitted to a linear mixture model. The search for the optimum was carried out using Numerical Optimisation of Design-Expert version 10 for maximising protein and minimising saturated fat. The linear model was suitable for explaining total protein and saturated fat variation with r^2^ of 0.50 and 0.51, respectively. Two final formulations were generated through the optimisation process: OS1, for maximising protein, with 15:30:55 MLP, MPMF, and FPMF, respectively, with protein projected at 12.41% and a desirable 0.867. The second formulation (OS2) maximised protein while minimising saturated fat with 15:55:30 MLP, MPMF, and FPMF, respectively, and a projected 11.84% protein and 1.25% saturated fat and desirability of 0.625.

### 2.4. Preparation of Composite Instant Meal Powders

Blended FPMF and MPMF, as per OS1 and OS2 formulations, were combined with one-part equivalent of water by weight in a stainless-steel pot. The cold paste was stirred gently, and another 2 to 3 parts of boiling water were added to the mixture. The warm paste was cooked on a hot plate stove at medium heat with consistent stirring to reach 80–85 °C and held for 12 min with periodic stirring. The cooked mixtures were then removed from the heat and allowed to cool to <50 °C before adding a corresponding amount of MLP and gentle mixing to aid uniform distribution. The final pastes were then transferred into stainless steel trays, frozen overnight, and into an ultra-freezer (SL 9002, Snijders Scientific, Tilburg, The Netherlands) before freeze-drying using a freeze dryer (Genesis SQ Super XL-70, SP Scientific, Warminster, PA, USA). The dried samples were milled using a universal cutting mill with a 0.75 mm sieve (Pulverisette 19, Fritsch, Idar-Oberstein, Germany) and refrigerated in sealed ziplock plastic containers until analysis.

### 2.5. Nutritional Analysis

Protein content was determined using the nitrogen analyser (TruSpec^®^ N, Leco Cor-poration, St. Joseph, MI, USA) using 6.25 as the nitrogen conversion factor. Amino acids analysis was carried out by derivatisation with AccQ-TagTM ultra-amino acid kit (Waters Corporation, Milford, MA, USA) and quantification using ultra-performance liquid chromatography (UPLC) with UV detection. Fat content was analysed as per the AOAC 996.06 method, sugars by the AOAC 982.14 method, and vitamin C by the AOAC 982.14 method [25]. Minerals were analysed using inductively coupled plasma optical emission spectroscopy (ICP-OES). Moisture content was assayed using a procedure based on AOAC Method 934.01 Air Oven Method [26].

### 2.6. Extraction of Phenolics

The samples were mixed with 1% HCL–methanol solution using a 1:10 (*v*/*v*) sample to the solvent ratio in 50 mL screw-cap tubes. Tubes were shaken gently to mix components adequately and left to stand overnight in a dark place. Samples were prepared in triplicates.

### 2.7. Total Phenolic Content

The Folin-Ciocalteu method was used to determine TPC as per Sadasivam and Manickam [27], with slight modifications. In 6 Eppendorf tubes, gallic acid standard stock solutions of 0, 20, 50, 100, 250, and 500 mg/L were diluted with 10% Ethanol. 25 μL of each different concentration of the standard and extracts were pipetted into the 96 healthy plates in triplicate after which 125 μL of the Folin-Coicalteau phenol reagent was then added and the mixture was left to stand for 5 min. Then 100 μL of 7.5% Na_2_CO_3_ solution was added to each well and the plate was left to stand for 2 h at room temperature. Absorbance in the wells was read at 750 nm using a spectrophotometer (Multiskan Spectrum, Thermo Electron Corp., Waltham, MA, USA). TPC was expressed in milligrams of gallic acid equivalent per 100 g of dry weight.

### 2.8. Oxygen Radical Absorbance Capacity (ORAC) Assay

The preparation and analysis procedures of samples for the ORAC assay were conducted as per ARL CPUT [28]. Trolox standard 500 µM stock solution was prepared by weighing 0.00625 g 6-Hydroxy-2,5,7,8-tetra-methylchroman-2-carboxylic acid in a 50 mL screw-cap tube and adding 50 mL phosphate buffer with Gilson pipetting aid, and mixed until dissolved. A 150 mg portion of Peroxyl radical, AAPH (2,2′-Azobis (2-methylpropionamidine) dihydrochloride was pre-weighed into a 15 mL screw cap tube. A 96-well plate reader was loaded with Trolox standards, control, and sample wells. A 10 µL portion of the fluorescein stock solution was added into 2 mL phosphate buffer in an Eppendorf tube and 240 µL of this solution was diluted in 15 mL phosphate buffer using a 15 mL screw cap tube. A 138 µL portion of this solution was pipetted with a multichannel pipette into each well of a black 96-microwell plate. A 6 mL portion of the phosphate buffer was added to the pre-weighed AAPH and mixed well until dissolved. A 50 µL portion of this solution was transferred using a multichannel pipette into each well. The multi-well plate was inserted into the fluorometer, with the excitation wavelength set at 485 nm, the emission wavelength at 530 nm, and the temperature at 37 °C before initiating analysis. Results were based on the principle that one ORAC unit is assigned as being the net protection area provided by 1 μM Trolox in the final concentration. The area under the curve for the sample was compared to the area under the curve for Trolox, and the result given was reported in Trolox equivalents per weight of the sample (μmole TE/100 g).

### 2.9. Pasting Properties

Pasting properties were characterised using the Rapid Visco Analyzer (RVA 4500, Perten Instruments, Macquarie Park, NSW, Australia). Approximately 3 g of sample was mixed with 25 mL of distilled water and heating cycles were set as follows. The sample was heated to 50 °C for 1 min, with subsequent heating to 95 °C over 3.42 min, and held for 2.5 min at 95 °C before cooling to 50 °C over 3.80 min and holding at this temperature for the final 2.5 min with a total analysis time adding up to 13 min. The following parameters were analyzed, peak viscosity (PV), trough viscosity (TV), breakdown viscosity (BV), final viscosity (FV), setback viscosity (SV), peak time (PT), and pasting temperature. All samples were analysed in triplicate.

### 2.10. Water Absorption Index and Water Solubility Index

Water absorption index (WAI) and water solubility index (WSI) were measured according to a method outlined by Obilana [29] as adapted from Anderson et al. [30] and Athar et al. [31]. WAI was measured by the dissolution of samples in warm distilled water before centrifugation and subsequent weighing of the gel yielded after pouring supernatant into an evaporating dish. WSI was carried out by evaporating supernatant from the centrifugal treatment of sample solution (using distilled water) where WSI was indicated by the weight of dissolved solids divided by the weight of dry solids expressed as a percentage. Calculations for WAI and WSI were carried out as per Equations (1) and (2) below.

(1) WAI (%)=(weight of sediments/weight of dry solids)×100

(2) WSI (%)=(weight of solids dissolved in supernatant/weight of dry solids)×100

### 2.11. Water Activity

Water activity (a_w_) was measured using a water activity meter (Rotronic HC2-a_w_, Rotronic AG, Bassersdorf, Switzerland).

### 2.12. Statistical Analysis

Analysis of variance (ANOVA) was used to determine the mean difference between treatments and compositions (at *p* = 0.05) and Duncan’s multiple ranges tests were employed to separate means where differences exist. Statistical analysis was performed in triplicate (*n* = 3) except for amino acids, sugars, and vitamins C which were conducted in duplicate (*n* = 2), using the IBM Statistical Package for Social Science (IBM SPSS, version 26, 2019).

## 3. Results and Discussion

### 3.1. Proximate Composition

The proximate composition of pearl millet (PM) and Moringa leaf powder (MLP) composite meal powders is indicated in Table 1.

Moisture content (MC) ranged from 4.15 to 4.50 g/100 g for the PM and MLP composite food powders OS1 and OS2, values that were substantially low compared to the 9.47 g/100 g of RPMF. The range of MC for the inputs was 2.89 to 7.87 g/100 g, with FPMF exhibiting a very low moisture content, possibly aided by freeze drying applied to render the fermented slurry into a powder. The low MC acquired on the composite powders OS1 and OS2 will favour keeping quality.

The ash content of both PM and MLP composite food powders was 2.93 g/100 g translating to a 75% increase from the 1.67 g/100 g of RPMF. El-Fatah et al. [14] and Nour and Ebrahim [15] yielded even higher ash content increases of 92% and 240% upon compositing MLP with cereal flours at ratios of 7.5% and 15%, respectively. The increase can be attributed to compositing PM with MLP whose ash content was 10.76 g/100 g. The overall impact of MLP on the ash content of PM could help alleviate mineral deficiencies in consumers.

The protein content of the PM and MLP composite food powders ranged from 12.59 to 13.51 g/100 g, translating to a maximum increase of 22% from the 11.12 g/100 g for RPMF. The two protein content results for OS1 (12.59 g/100 g) and OS2 (13.51 g/100 g) were higher than the projected 12.41 g/100 g and 11.85 g/100 g, quantities from the optimisation process, respectively. The significant (*p* ≤ 0.05) protein increase can be attributed to MLP with a high protein content of 26.32 g/100 g. The protein content of the PM and MLP composite food powders was above the average protein content for selected common South African market instant cereal powders ranging from 7.30 to 16.50 g/100 g as reported by Wiles [32]. The elevated protein content of PM could help alleviate protein-energy malnutrition for communities that rely on PM as a staple food.

The fat content of the PM and MLP composite food powders ranged from 3.89 to 6.59 g/100 g, translating to a 25% increase and 26% decrease in the fat content of OS1 & OS2, respectively, compared to RPMF at 5.28 g/100 g. OS1 had a higher proportion of saturated fat at 2.43 g/100 g compared to the 1.54 g/100 g observed in OS2 owing to the optimisation process where the constraint of minimizing saturated fat was employed. Malting led to a significant (*p* ≤ 0.05) reduction (18%) in the fat content of PM, a result in line with the 12% and 26% reductions observed by Adebiyi et al. [33] and Embashu and Natanga [34], respectively, with the former attributing this to lipid breakdown during malting. MLP’s fat content (2.71 g/100 g) was quite lower than the 4.50 g/100 g and 5.75 g/100 g reported by Offor et al. [35] and Penalver et al. [36], possibly due to varietal differences.

Compositing malted and fermented PM with MLP led to a significant (*p* ≤ 0.05) increase in the total sugar content of the PM and MLP composite food powders with a range of. 6.00 to 7.40 g/100 g compared to RPMF’s 1.10 g/100 g. MPMF and MLP contributed to elevated total sugar as found in OS2 with maximum contents of the two components. The elevated quantity of sugars in malted PM could be attributed to starch hydrolysis by endogenous enzymes such as amylase to produce free sugars. The fermented PM showed a similarly elevated total sugar content as starch was hydrolysed into simple sugars before subsequent conversion to organic acids by lactic acid bacteria.

The Vitamin C content of PM and MLP composite meal powders is presented in Table 2.

The vitamin C content of the composite meal powders ranged between 33.0 and 49.3 mg/100 g. These values were notably higher than the trace amounts in PM (not exceeding 1 mg/100 g) reported by Nambiar et al. [37] and Pei et al. [38]. On the other hand, Abbas et al. [39] reported up to 86 mg/100 g of vitamin C in MLP. The elevated Vitamin C content in OS2 may be attributed to its biosynthesis from simple sugars (glucose, mannose, and galactose) accumulated during the enzymatic hydrolysis of starch, as affirmed by Kazi et al. [40].

### 3.2. Amino Acid Content

Table 3 shows the amino acid content of PM and MLP composite food powders alongside RPMF, MPMF, FPMF, and MLP. Of the nine essential amino acids, eight were found with tryptophan being the exception. The lysine content of the composite food powders ranged from 0.45 to 0.55 g/100 g with RPMF at 0.45 g/100 g.

Overall, there appeared to be no significant (*p* ≤ 0.05) increase in lysine content with the addition of MLP at a maximum of 15%, although OS1 showed a 22% increase. Processing of PM (malting and fermentation) reduced the lysine content by up to 33%, a trend that may have impacted the overall augmentation significance of MLP which had a significantly (*p* ≤ 0.05) higher value of 1.00 g/100 g. The reduction of lysine during fermentation was unexpected but no different from the findings presented by Osman [41]. The lysine content of MLP was found to be very similar to Cattan et al. [42] but slightly lower than 1.54 g/100 g reported by Juhaimi et al. [43]. There was a 50% increase in the sulphur-containing methionine from 0.40 g/100 g (RPMF) to 0.60 g/100 g for both PM and MLP composite food powders. Isoleucine and threonine increased by up to 75% and 100% respectively in the PM and MLP composites. Other essential amino acids comprising leucine, phenylalanine, and valine also significantly (*p* ≤ 0.05) increased particularly in OS1. The differences in amino acid contents of OS1 and OS2, with OS1 showing a superior profile, may be explained by the higher protein content observed in OS1 due to its elevated FMPF content.

### 3.3. Mineral Composition

Table 4 shows the mineral composition of PM and MLP composite food powders, alongside RPMF, MPMF, FPMF, and MLP. The calcium (Ca) content of the PM and MLP composite food powders ranged between 381.5 to 414.8 mg/100 g translating to over 1200% increase (from the 30.8 mg/100 g in RPMF). This may be attributed to the high Ca content (1477.0 mg/100 g) of MLP. The iron (Fe) content averaged 7.22 mg/100 g translating to over 100% increase owing to the high Fe content of MLP. MLP’s Fe content of 37.52 mg/100 g was higher than the 25.14 mg/100 g reported by Penalver et al. [36]. The magnesium (Mg) content ranged between 148.7 and 152.8 mg/100 g translating to over 50% increase in food composite powders when compared to RPM, owing to MLP’s figure of 282.0 mg/100 g. The Mg content of MLP was higher than the 233.5 mg/100 g stated by Nour and Ibrahim [15] but lower than the 301.1 mg/100 g reported by Penalver et al. [36].

Other minerals comprising manganese (Mn), potassium (K), and sodium (Na) increased by up to 41%, 10%, and 75%, respectively, with increases in Mn and K attributed to their higher proportions in MLP whilst Na boost came from malted PM as introduced by the soaking process. Although not significant (*p* ≤ 0.05), reductions were observed in the copper and zinc content of PM and MLP composite food powders.

### 3.4. Functional Properties

#### 3.4.1. Pasting Properties

The pasting properties of PM and MLP composite food powders are summarised in Table 5.

The peak viscosity of the PM and MLP composite food powders OS1 and OS2 ranged from 27.67 to 45.67 Cp, the final viscosity ranged between 46.33 and 63.67 Cp and the peak viscosity ranged between 46.33 to 48.00 Cp. Trough viscosity, breakdown viscosity, and setback viscosity ranged from 23.00 to 30.33 Cp, 4.67 to 15.33 Cp and, 23.33 to 33.33 Cp, respectively. Fermentation increased the viscosity of PM significantly (*p* ≤ 0.05) as also reported by Akinola et al. [44] and Adepehin et al. [45], with both studies citing structural starch granule modifications such as swelling. Conversely, malting significantly decreased the viscosity of PM, as observed by Obilana [29] who attributed the trend to the degradation of starch by amylases.

The low viscosity of the composite food powders, influenced by MPMF and MLP, indicates a reduced ability to form a viscous paste, which is associated with higher nutrient density Fili et al. [46] and Awolu et al. [47]. This was evident during the cooking of the raw composite PM and MLP flours for numerical optimisation whereby recipes with higher proportions of malted flour required 33% less water to achieve desired simmering consistency compared to recipes with more fermented flour. Pelembe et al. [48] further postulated that the decrease in viscosity could be advantageous for infants or anyone who requires spoonable viscosity, with retention of high nutrient or energy density.

#### 3.4.2. Water Interaction Properties

The water interaction properties of PM and MLP composite food powders, alongside RPMF, MPMF, FPMF, and MLP are summarised in Table 6.

The water absorption index (WAI) ranged from 418.90 to 481.94% for the PM and MLP composite food powders. The WAI values of RPMF and MPMF were similar to the findings of Obadina et al. [49]. WAI has been ascribed to amylose/amylopectin ratios in the flour with higher amylose directly correlated to WAI Awolu et al. [50]. The higher WAI values of PM and MLP composite flours may then be attributed to MLP, which had a high WAI (423.46%) ascribed to its high crude fibre peaking at over 10% [15]. Moreover, the gelatinisation of starch, as postulated by Obadina et al. [49], results in more open or enlarged starch granules that readily absorb water and paste therefore rendering the cooked composite flours more suitable for cold to warm water reconstitution to prepare instant beverage or porridge meals.

Water solubility index (WSI), ranged from 21.81 to 32.98% for the PM and MLP composite food powders. The WSI values for RPMF (7.71%) and MPMF (13.10%) were comparable to those of Obilana [29] and Obadina et al. [49], with RPMF values of 5.14% and 5.13% and, MPM values of 12.16% and 12.62%, respectively. The aforementioned authors similarly reported a significant increase in WSI as a result of malting PM, with Obadina et al. [29] ascribing this to the depolymerisation of starch due to enzymatic action in crystalline regions of PM leading to elevated hygroscopicity. Fermentation of PM also resulted in a significant (*p* ≤ 0.05) increase in WSI a trend similar to Simwaka et al. [50] and Onweluzo et al. [51] who attributed this to hydrolysis of high molecular weight carbohydrates and proteins to simpler and more soluble forms during fermentation. WSI serves as an indication of starch degradation and dextrinisation and ultimately the amount of soluble and digestible materials in PM, therefore, a higher WSI would be desirable for flours used to prepare instant meals by reconstituting with water.

Water activity (a_w_) for the PM and MLP composite food flours ranged between 0.3267 to 0.3300, significantly lower than RPMF at 0.5100. Moisture content and water activity are generally related to keeping quality or shelf life, with flours ideally required to have low values for the two parameters. A combination of low moisture content and low water activity limits available water for microbial activity and consequently spoilage, imparting stability and reducing chances of rancidity and ultimately a longer shelf life product [44,47].

#### 3.4.3. Total Phenolic Content and Antioxidant Properties

Table 7 illustrates the total phenolic content and antioxidant properties of the PM and MLP composites alongside RPMF, and inputs MPMF, FPMF, and MLP.

Total phenolic content (TPC), and oxygen radical absorbance capacity (ORAC) for the composite food powders ranged from 477.44 to 513.33 mg GAE/100 g and 11,048.90 to 11,700.11 μmol TE/100 g, respectively. Both TPC and ORAC values of PM and MLP composite flours were significantly (*p* ≤ 0.05) superior to those of RPM. This can be attributed to the addition of MLP that presented significantly (*p* ≤ 0.05) higher values on both parameters than FPMF and MPMF. The TPC value for MLP, 1482.56 mg GAE/100 g was, however, lower than the 3290 mg GAE/100 g reported by Penalver et al. [36].

Despite some phenolic compounds, particularly phytates from cereal grains, being known to present anti-nutritional properties, the greater portion of these compounds detected in the PM and MLP composites were of MLP origin. MLP phenolic compounds have, on the other hand, been reported to play key roles in the human body comprising anti-carcinogenic, immunomodulatory, anti-diabetic, and antiatherogenic functions [52,53,54].

## 4. Conclusions

The protein content results obtained from the analysis of the two PM and MLP composite food powders (OS1 and OS2) were aligned with projected outcomes from the mixture design optimisation process. There were no significant differences in the ash content and protein content of OS1 and OS2 as both formulations had maximum proportions of MLP which contributed most of the minerals and proteins. The total fat content was lower in OS1 than in OS2 and therefore aligned with the optimisation constraints projections. These results indicated the effectiveness of mixture design in determining the optimum recipe for maximising the protein content and minimising saturated fats in the composite food powders. Both OS1 and OS2 indicated improvements in pasting properties as viscosity was reduced significantly (*p* ≤ 0.05) mostly owing to malting and the addition of MLP. *Moringa oleifera* leaf powder showed great effectiveness as a food fortificant with the significant (*p* ≤ 0.05). increases observed in protein and ash content on the PM and MLP composite food powders. Fermentation also led to a significant (*p* ≤ 0.05) increase in the protein content, a result not achieved through malting. These outcomes may help alleviate protein-energy malnutrition and mineral deficiencies in communities that rely on pearl millet cereal foods. Malting improved the PM’s pasting properties by reducing viscosity, a result associated with a higher nutrient density in the cooking of gruels as less water is required to achieve desired consistencies for consumption. The significant increase in phenolics observed as a result of the addition of MLP extended benefits to the potential impartation of nutraceutical benefits associated with MLP’s phytochemicals.

## Figures and Tables

**Table 1:** Proximate composition of PM and MLP composite food powders ^1^.

			Proximate Composition (g/100 g)		
Samples	Moisture	Ash	Protein	Total Fat	Total Sugars
RPMF	9.47 ± 0.04 ^a^	1.67 ± 0.02 ^a^	11.12 ± 0.30 ^a^	5.28 ± 0.41 ^a^	1.10 ± 9.27 ^a^
OS1	4.15 ± 0.14 ^b^	2.93 ± 0.01 ^b^	13.51 ± 0.18 ^b^	6.59 ± 0.48 ^b^	6.00 ± 7.05 ^b^
OS2	4.50 ± 0.10 ^c^	2.93 ± 0.02 ^b^	12.59 ± 0.27 ^b^	3.89 ± 0.34 ^c^	7.40 ± 4.80 ^c^
MPMF	7.87 ± 0.06 ^d^	1.59 ± 0.08 ^a^	9.98 ± 0.43 ^c^	4.33 ± 0.88 ^cd^	3.50 ± 4.80 ^d^
FPMF	2.89 ± 0.04 ^e^	1.61± 0.04 ^a^	12.40± 0.33 ^b^	4.88± 0.22 ^ad^	2.00 ± 4.80 ^e^
MLP	7.11 ± 0.12 ^f^	10.76 ± 0.13 ^c^	26.32 ± 1.25 ^d^	2.71 ± 0.29 ^e^	4.70 ± 4.80 ^f^

^1^ Values are mean ± standard deviation. Means with different superscripts in each column differ significantly (*p* ≤ 0.05). RPMF—Raw pearl millet flour, MPMF—Malted pearl millet flour, FPMF—Fermented pearl millet flour, MLP—Moringa leaf powder, OS1—15% MLP, 30% MPMF, 55% FPMF, OS2—15% MLP, 55% MPMF, 30% FPM.

**Table 2:** Vitamin C content of PM and MLP composite meal powders ^1^.

	OS1	OS2
Vitamin C	33.0 ± 0.36 ^a^	49.3 ± 0.14 ^b^

^1^ Values are mean ± standard deviation. Means with different superscripts in each row differ significantly (*p* ≤ 0.05), OS1—15% MLP, 30% MPMF, 55% FPMF, OS2—15% MLP, 55% MPMF, 30% FPMF.

**Table 3:** The amino acid content of PM and MLP composite food powders (g/100 g) ^1^.

Amino Acids	RPMF	OS1	OS2	MPMF	FPMF	MLP
Arginine	0.55 ± 0.07 ^a^	0.85 ± 0.35 ^ab^	0.70 ± 0.14 ^a^	0.50 ± 0.00 ^a^	0.55 ± 0.07 ^a^	1.60 ± 0.71 ^b^
Histidine	0.25 ± 0.07 ^a^	0.35 ± 0.07 ^ab^	0.35 ± 0.07 ^ab^	0.30 ± 0.00 ^a^	0.30 ± 0.00 ^a^	0.50 ± 0.14 ^b^
Isoleucine	0.40 ± 0.00 ^a^	0.70 ± 0.28 ^a^	0.55 ± 0.07 ^a^	0.40 ± 0.00 ^a^	0.50 ± 00 ^a^	1.10 ± 0.00 ^b^
Leucine	0.95 ± 0.07 ^a^	1.60 ± 0.42 ^b^	1.15 ± 0.07 ^a^	0.85 ± 0.07 ^a^	1.05 ± 0.70 ^a^	2.30 ± 0.00 ^c^
Lysine	0.45 ± 0.35 ^ab^	0.55 ± 0.07 ^ab^	0.45 ± 0.35 ^ab^	0.35 ± 0.21 ^a^	0.30 ± 0.14 ^a^	1.00 ± 0.28 ^b^
Methionine	0.40 ± 0.14 ^a^	0.60 ± 0.28 ^a^	0.60 ± 0.28 ^a^	0.25 ± 0.07 ^a^	0.30 ± 0.14 ^a^	0.30 ± 0.14 ^a^
Phenylalanine	0.90 ± 0.28 ^a^	1.85 ± 0.07 ^bc^	1.10 ± 0.14 ^ab^	0.90 ± 0.28 ^a^	1.05 ± 0.49 ^ab^	2.60 ± 0.57 ^c^
Threonine	0.45 ± 0.07 ^a^	0.90 ± 0.42 ^ab^	0.70 ± 0.14 ^a^	0.50 ± 0.00 ^a^	0.55 ± 0.07 ^a^	1.50± 0.40 ^b^
Tyrosine	0.45 ± 0.35 ^a^	0.70 ± 0.28 ^a^	0.65 ± 0.21 ^a^	0.40 ± 0.00 ^a^	0.45 ± 0.07 ^a^	1.75 ± 0.07 ^b^
Valine	0.50 ± 0.00 ^a^	0.85 ± 0.21 ^b^	0.60 ± 0.00 ^a^	0.45 ± 0.07 ^a^	0.55 ± 0.07 ^a^	1.35 ± 0.07 ^c^
Alanine	0.80 ± 0.00 ^a^	1.20 ± 0.28 ^b^	0.90 ± 0.00 ^ab^	0.75± 0.07 ^a^	0.90 ± 0.00 ^ab^	1.85 ± 0.07 ^c^
Asparagine	0.75 ± 0.07 ^a^	1.30 ± 0.42 ^a^	1.00 ± 0.00 ^a^	0.95 ± 0.07 ^a^	0.80 ± 0.00 ^a^	2.65 ± 0.35 ^b^
Serine	0.60 ± 0.00 ^a^	0.95 ± 0.35 ^a^	0.70 ± 0.14 ^a^	0.60 ± 0.00 ^a^	0.55 ± 0.21 ^a^	1.55 ± 0.07 ^b^
Glutamate	1.75 ± 0.21 ^a^	2.45 ± 0.78 ^ab^	1.85 ± 0.07 ^a^	1.70 ± 0.14 ^a^	1.85 ± 0.07 ^a^	3.35 ± 0.50 ^b^
Proline	0.50 ± 0.14 ^a^	0.80 ± 0.42 ^a^	0.55 ± 0.21 ^a^	0.45 ± 0.07 ^a^	0.50 ± 0.14 ^a^	1.00 ± 0.14 ^a^

^1^ Values are mean ± standard deviation. Means with different superscripts in each row differ significantly (*p* ≤ 0.05) RPMF—Raw pearl millet flour, MPMF—Malted pearl millet flour, FPMF—Fermented pearl millet flour, MLP—Moringa leaf powder, OS1—15% MLP, 30% MPMF, 55% FPMF, OS2—15% MLP, 55% MPMF, 30% FPM.

**Table 4:** The mineral content of PM and MLP composite food powders ^1^.

Minerals	RPMF	OS1	OS2	MPMF	FPMF	MLP
Ca	30.8 ± 0.78 ^a^	414.8 ± 13.4 ^b^	381.5 ± 14.4 ^b^	31.0 ± 0.41 ^a^	26.7 ± 1.40 ^a^	1477.0 ± 46.97 ^c^
Cu	0.48 ± 0.01 ^a^	0.40 ± 0.01 ^a^	0.40 ± 0.01 ^a^	0.56 ± 0.08 ^b^	0.52 ± 0.00 ^b^	0.87 ± 0.08 ^c^
Fe	3.51 ± 0.04 ^a^	7.23 ± 0.46 ^a^	7.20 ± 0.36 ^a^	3.28 ± 0.53 ^a^	2.58± 0.06 ^a^	37.52 ± 6.10 ^b^
Mg	98.5 ± 0.86 ^a^	152.8 ± 1.81 ^b^	148.7 ± 1.60 ^b^	89.4 ± 1.84 ^c^	79.2 ± 2.91 ^d^	282.0 ± 9.29 ^e^
Mn	1.21 ± 0.35 ^a^	1.71 ± 0.04 ^b^	1.31 ± 0.01 ^b^	1.09 ± 0.09 ^c^	0.95 ± 0.04 ^c^	3.19 ± 0.25 ^d^
K	435.9 ± 8.37 ^a^	479.8 ± 1.23 ^b^	450.1 ± 5.08 ^a^	343.6 ± 4.26 ^c^	368.3 ± 24.00 ^c^	897.7 ± 29.61 ^d^
Na	42.8 ± 1.54 ^ae^	61.4 ± 1.64 ^b^	74.9 ± 0.57 ^c^	115.9 ± 9.14 ^d^	39.4 ± 4.78 ^a^	55.11 ± 13.48 ^e^
Zn	2.14 ± 0.16 ^a^	1.86 ± 0.06 ^a^	1.94 ± 0.14 ^a^	2.15 ± 0.39 ^a^	1.83 ± 0.12 ^a^	3.27 ± 0.95 ^b^

^1^ Values are mean ± standard deviation. Means with different superscripts in each row differ significantly (*p* ≤ 0.05) RPMF—Raw pearl millet flour, MPMF—Malted pearl millet flour, FPMF—Fermented pearl millet flour, MLP—Moringa leaf powder, OS1—15% MLP, 30% MPMF, 55% FPMF, OS2—15% MLP, 55% MPMF, 30% FPMF.

**Table 5:** Pasting properties of PM and MLP composite food powders ^1^.

Minerals	Peak Viscosity (cP)	Trough Viscosity (cP)	Breakdown Viscosity (cP)	Final Viscosity (cP)	Setback Viscosity (cP)	Pasting Temp. °C
RPMF	281.67 ± 0.58 ^a^	260.00 ± 2.00 ^a^	21.67 ± 2.08 ^a^	866.67± 17.62 ^a^	606.67 ± 15.63 ^a^	89.93 ± 0.03
OS1	45.67± 2.08 ^b^	30.33 ± 0.58 ^b^	15.33 ± 1.53 ^b^	63.67 ± 1.15 ^b^	33.33 ± 0.58 ^b^	ND
OS2	27.67 ± 1.52 ^b^	23.00 ± 0.00 ^b^	4.67 ± 1.53 ^c^	46.33 ± 1.15 ^b^	23.33 ± 1.15 ^b^	ND
MPMF	34.67 ± 2.89 ^b^	23.67 ± 1.15 ^b^	11.00 ± 1.73 ^bc^	48.00 ± 2.65 ^b^	24.33 ± 1.53 ^b^	ND
FPMF	830.00 ± 83.26 ^c^	803.00 ± 75.90 ^c^	27.00 ± 7.81 ^a^	1435.33 ± 136.08 ^c^	632.33 ± 60.58 ^a^	87.85 ± 0.57
MLP	27.00 ± 3.61 ^b^	16.33 ± 3.51 ^b^	10.66 ± 0.58 ^bc^	30.00 ± 3.61 ^b^	13.67 ± 0.58 ^b^	ND

^1^ Values are mean ± standard deviation. Means with a different superscript in each column differ significantly (*p* ≤ 0.05), ND—Not Determined, RPMF—Raw pearl millet flour, MPMF—Malted pearl millet flour, FPMF—Fermented pearl millet flour, MLP—Moringa leaf powder, OS1—15% MPL, 30% MPMF, 55% FPMF, OS2—15% MPLF, 55% MPMF, 30% FPMF.

**Table 6:** Water interaction properties of PM and MLP composite meal powders ^1^.

Samples	WAI (%)	WSI (%)	Water Activity (a_w_)
RPMF	249.40 ± 10.14 ^a^	7.71 ± 0.93 ^a^	0.5100 ± 0.01 ^a^
OS1	481.94 ± 7.39 ^b^	21.81 ± 1.85 ^b^	0.3300 ± 0.00 ^b^
OS2	418.90 ± 27.56 ^c^	32.98 ± 3.58 ^c^	0.3267 ± 2.00 ^b^
MPMF	272.78 ± 13.23 ^a^	13.10 ± 1.54 ^d^	0.4200. ± 0.01 ^c^
FPMF	248.94 ± 3.53 ^a^	13.44 ± 1.42 ^d^	0.1967 ± 0.06 ^d^
MLP	423.46 ± 12.96 ^c^	30.03 ± 2.76 ^c^	0.5133 ± 0.01 ^a^

^1^ Values are mean ± standard deviation. Means with different superscripts in each column differ significantly (*p* ≤ 0.05) WAI—Water absorption index, WSI—Water solubility index, RPMF—Raw pearl millet flour, MPMF—Malted pearl millet flour, FPMF—Fermented pearl millet flour, MLP—Moringa leaf powder, OS1—15% MLP, 30% MPMF, 55% FPMF, OS2—15% MLP, 55% MPMF, 30% FPMF.

**Table 7:** Phenolic content and antioxidant properties of PM and MLP composite meal powders ^1^.

Samples	TPC (mg GAE/100 g)	ORAC (μmol TE/100 g)
RPMF	284.62 ± 9.27 ^a^	8127.88 ± 709.05 ^a^
OS1	477.44 ± 4.62 ^b^	11,048.90 ± 553.28 ^b^
OS2	513.33 ± 23.50 ^b^	11,700.11 ± 1051.00 ^b^
MPMF	315.90 ± 4.07 ^a,c^	9334.97 ± 188.15 ^a^
FPMF	342.57 ± 29.60 ^c^	8959.10 ± 46.56 ^a^
MLP	1482.56 ± 54.00 ^d^	19,779.54 ± 264.86 ^c^

^1^ Values are mean ± standard deviation. Means with different superscripts in each column differ significantly (*p* ≤ 0.05). TPC—Total phenolic content (GAE—Gallic acid equivalent), ORAC—Oxygen radical absorbance capacity (TE—Trolox equivalent), RPMF—Raw pearl millet flour, MPMF—Malted pearl millet flour, FPMF—Fermented pearl millet flour, MLP—Moringa leaf powder, OS1—15% MLP, 30% MPMF, 55% FPMF, OS2—15% MLP, 55% MPMF, 30% FPMF.

## Data Availability

The data presented in this study are available on request from the corresponding author. The data are not publicly available due to privacy restrictions.
